# Inequities in utilization of maternal health interventions in Namibia: implications for progress towards MDG 5 targets

**DOI:** 10.1186/1475-9276-9-16

**Published:** 2010-06-12

**Authors:** Eyob Zere, Prosper Tumusiime, Oladapo Walker, Joses Kirigia, Chris Mwikisa, Thomas Mbeeli

**Affiliations:** 1 Inter-country Support Team for Eastern and Southern Africa, World Health Organization, Regional Office for Africa, Harare, Zimbabwe; 2 World Health Organization, Regional Office for Africa, Brazzaville, Congo; 3 Ministry of Health and Social Services, Windhoek, Namibia

## Abstract

**Background:**

Inequities in the utilization of maternal health services impede progress towards the MDG 5 target of reducing the maternal mortality ratio by three quarters, between 1990 and 2015. In Namibia, despite increasing investments in the health sector, the maternal mortality ratio has increased from 271 per 100,000 live births in the period 1991-2000 to 449 per 100,000 live births in 1998-2007. Monitoring equity in the use of maternal health services is important to target scarce resources to those with more need and expedite the progress towards the MDG 5 target. The objective of this study is to measure socio-economic inequalities in access to maternal health services and propose recommendations relevant for policy and planning.

**Methods:**

Data from the Namibia Demographic and Health Survey 2006-07 are analyzed for inequities in the utilization of maternal health. In measuring the inequities, rate-ratios, concentration curves and concentration indices are used.

**Results:**

Regions with relatively high human development index have the highest rates of delivery by skilled health service providers. The rate of caesarean section in women with post secondary education is about seven times that of women with no education. Women in urban areas are delivered by skilled providers 30% more than their rural counterparts. The rich use the public health facilities 30% more than the poor for child delivery.

**Conclusion:**

Most of the indicators such as delivery by trained health providers, delivery by caesarean section and postnatal care show inequities favoring the most educated, urban areas, regions with high human development indices and the wealthy. In the presence of inequities, it is difficult to achieve a significant reduction in the maternal mortality ratio needed to realize the MDG 5 targets so long as a large segment of society has inadequate access to essential maternal health services and other basic social services. Addressing inequities in access to maternal health services should not only be seen as a health systems issue. The social determinants of health have to be tackled through multi-sectoral approaches in line with the principles of Primary Health Care and the recommendations of the Commission on Social Determinants of Health.

## Introduction

There has been a heightened concern for socio-economic inequalities in health and access to health care and the social determinants of health as countries progress towards the target date for achieving the Millennium Development Goals (MDGs) [1]. There is increasing evidence demonstrating that the poor and marginalized segments of society have the worst health status and access to health enhancing interventions [2-5]. Access to health care still follows the inverse care law, where the wealthiest that have relatively less need for health care consume more of it [6].

Equity in maternal health outcomes and access to maternal health interventions has been on top of the equity agenda, as maternal health is an important component of the MDGs [1]. One of the targets of MDG 5 is the reduction of maternal mortality by three quarters, between 1990 and 2015, and is monitored by two indicators: (i) maternal mortality ratio; and (ii) proportion of births by skilled health personnel [7].

Monitoring equity in maternal health and health services is important in order to target scarce public resources to those with more need and enhance the progress towards achieving the global targets. A goal that is defined in terms of population averages can result in inequities if efforts are not made to reach the poor and vulnerable. Furthermore, it is very difficult to achieve the health MDG targets without addressing inequities in health and health care, since it is among the poorest groups that the indicators are unfavourable and that there is a significant potential for improvement [1].

The maternal mortality ratio in Namibia has increased from 271 per 100,000 live births in the period 1991-2000 to 449 per 100,000 live births in the period 1998-2007 [8]. Although it is important to disaggregate the maternal mortality ratio by some critical social determinants such as education and geographical location (e.g. region), this is not possible in the current case, as the DHS report does not provide this due to smallness of the sample size used.

The increase in maternal mortality has been witnessed despite a relatively good and increasing coverage of maternal health interventions such as antenatal care and delivery by skilled health workers [8]. Thus, to reverse the trend and eventually achieve the target of reducing maternal mortality, it is necessary to analyze the situation using the equity lens and endeavour towards reaching those that are lagging behind in terms of the health outcomes and uptake of essential maternal health interventions.

The objective of this study is therefore to identify and measure socio-economic inequalities in access to maternal health services using data from the Namibia Demographic and Health Survey (NDHS) and propose recommendations relevant for policy and planning.

### Brief country profile

Namibia is a country in the South Western part of Africa covering a land area of 824,000 square kilometers. According to the 2001 population and housing census, the population was about 1.8 million with an intercensal growth rate of 2.6% per annum [9].

Namibia is a lower middle income country [10]. The human Development Index (HDI) was 0.686 (medium human development) in 2007 and the country ranked 128^th ^out of 182 countries on the HDI scale [11]. However, this average figure masks the fact that there are certain segments of the Namibian society whose HDIs may fall under the low human development category [12]. A Gini index of 60.3 [13] indicates that the country is one of those with the highest income inequality in the world. Table 1 below presents data on selected health and development indicators [8,10,14,15].

**Table 1:** Namibia: selected health and development indicators

Indicator	Value
Life expectancy at birth (years, 2007)	59
Infant mortality rate per 1000 live births (2002-2006)	46
Under-five mortality rate per 1000 live births (2002-2006)	69
Total fertility rate (2006-2007)	3.6
Maternal mortality ratio per 100,000 live births (1998-2007)	449
Percentage of pregnant women receiving antenatal care from a skilled provider (2006-2007) (%)	94.6
Percentage of child deliveries by a skilled provider (2006-2007) (%)	81.4
Adult (15-49 years) HIV prevalence rate (2008) (%)	15.3
Gross national income (GNI) per capita in US$ (2007)	3,360
Health expenditure per capita (real 2006 US$)	276
Physicians per 10,1000 population (2000-2007)	3
Nursing and midwifery personnel per 10,000 population (2000-2007)	31
Hospital beds per 10,000 population (2000-2008)	33

Sources [8,10,14,15]

## Data and methods

### Conceptual framework

Equity may be defined from three perspectives: equity in health; equity in health provision; and equity in health financing. The focus of this study is equity in health provision. Following Whitehead's seminal definition [16], equity in health is defined as the absence of systematic inequalities in health (or in the major social determinants of health) among people that have different positions in a social hierarchy. Maldistribution of health care is one of the social determinants of health. Equity in health care provision may therefore be defined as the absence of socio-economic inequalities in access to available maternal health services [4,17,18].

Three steps are followed in measuring equity in access to maternal health services: (i) identification of the intervention whose distribution is to be measured (e.g. antenatal care); (ii) classification of the population (women) by an indicator of socio-economic status; and (iii) measuring/quantifying the degree of inequality. The social stratifiers used in the current study are household wealth as derived from asset indices; mother's education; place of residence (urban/rural); and geographical region as there is a spatial dimension to the distribution of poverty and human development in the country [12].

### Data sources and variables

The study uses information from the Namibia demographic and health survey 2006-2007.

The variables measured include antenatal and postnatal checkup, delivery and caesarean section.

### Data analysis

**Figure 1:**
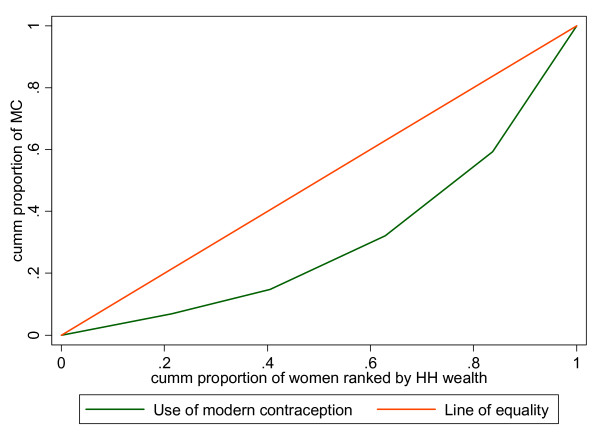
**Concentration curve: use of modern contraception**.

The health concentration curve and index as well as rate-ratios are used in measuring inequities. The concentration curve plots the cumulative proportion of the pregnant and parturient women ranked by their household wealth against the cumulative proportion of the health care variable (e.g. antenatal care). In other words, concentration curves capture the use of health interventions, cumulatively for each wealth quintile [19]. To demonstrate the use of the concentration curve, the case of use of modern contraception by women is presented in Figure 1 using hypothetical data.

In the absence of wealth-related inequality in the use of modern contraception, the concentration curve overlaps with the diagonal line (line of equality). This implies that there are no inequities in the use of modern contraception. A concentration curve that lies below the diagonal line signifies the presence of inequities favouring the rich, i.e. there is a disproportionately higher rate of contraceptive use among the wealthiest groups than the poorest. When the concentration curve lies above the line of equality, there is inequity favouring the poor, i.e. more women in the poorest group use modern contraception compared to the wealthiest. The degree of inequity becomes more when the concentration curve is further from the line of equality.

Concentration curves are a good graphical illustration to identify whether socioeconomic inequality in some health sector variable exists and whether it is more pronounced at one point in time than another or in one country than another. However, they don't quantify the magnitude of inequality for convenient comparison across many time periods, countries, regions, or whatever may be chosen for comparison.

The concentration index that is computed from the concentration curve quantifies the degree of socio-economic inequality in a health variable and assumes values between -1 and +1. Its value is negative when the concentration curve is above the diagonal and positive when the curve is below the diagonal. In the absence of inequities (the concentration curve coinciding with the diagonal), the value of the concentration index is zero.

From grouped data, the concentration index (C) is computed in a spreadsheet programme using the formula [20]:

Where *p *is the cumulative percent of the sample ranked by economic status (in this case the cumulative percentage of pregnant/parturient mothers ranked by wealth);

*L(p) *is the corresponding concentration curve ordinate (e.g. cumulative percentage of caesarean section); and

*T *is the number of socioeconomic groups (in this case *T *= 5, as there are five wealth quintiles)

To test for the statistical significance of the concentration index, standard errors can be computed using the formula given in Kakwani *et al *[21]. Data is analyzed using STATA 10 statistical software.

## Results

The socio-economic stratifiers used in this study are Geographical location (administrative region), Place of residence (urban/rural), mother's education and wealth quintile. Presentation of the results will therefore follow this sequence.

### 1. Geographical location

**Figure 2:**
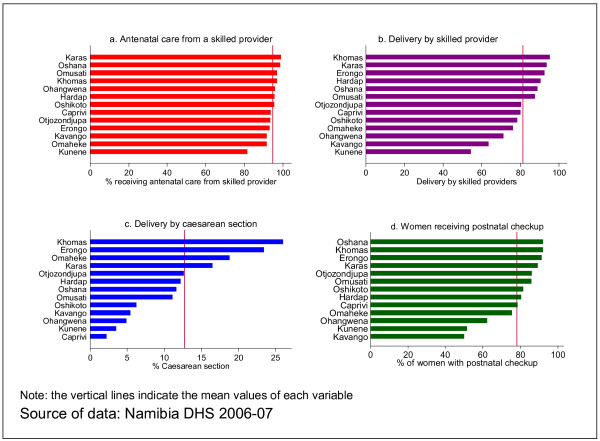
**utilization of interventions by region**.

No significant differences are observed in the regional distribution of the provision of antenatal care by a skilled provider. However, there is a remarkable difference in delivery by skilled health workers, caesarean section and postnatal checkup as can be observed from the Figure 2 below.

For nearly more than half of the regions, delivery by skilled health providers and caesarean section fall short of the population averages. Khomas, the region where the capital city is located has the highest rates of delivery by skilled attendants and caesarean section. There are 70% more deliveries by trained health workers in Khomas than in Kunene region, which has the lowest rate (54.4%). The disparity with respect to caesarean section is very striking. The rate in Khomas region (26%) is about thirteen times that of the Caprivi region, which has the lowest rate of caesarean section (2.2%).

**Figure 3:**
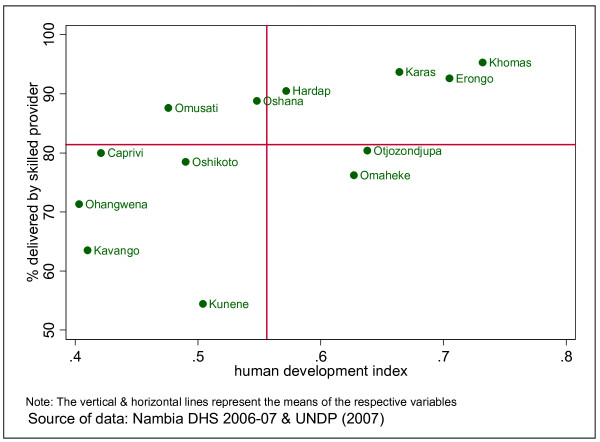
**HDI vs. delivery by skilled providers**.

The geographical inequality seems to follow the distribution of the social determinants of health. Those regions that have the lowest human development index seem to have the lowest uptake of the basic maternal health interventions. The case of delivery by skilled health personnel, which is an important indicator of MDG 5 presented in Figure 3 attests to this.

The majority of regions with HDI that is less than the national average have the lowest rates of delivery by skilled health providers (*r = 0.6270; P < 0.05 *implying a moderately strong positive correlation). It is known that the HDI is a composite indicator comprising Income per capita, life expectancy at birth, adult literacy and gross school enrolment ratio. Hence, the distribution of uptake of maternal health interventions is also related to those components of the HDI, which are largely outside the health sector.

### 2. Mother's education

**Figure 4:**
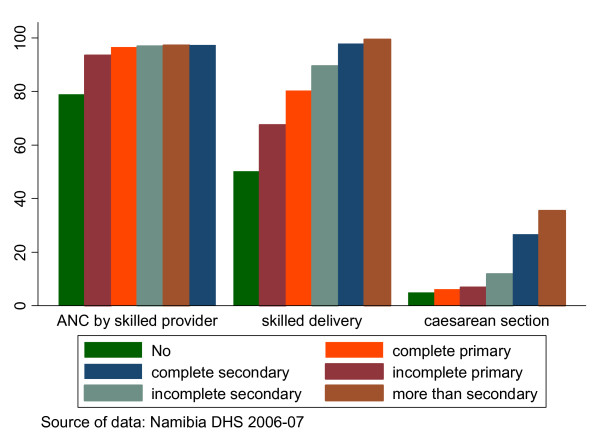
**Uptake of interventions by mother's education**.

As seen above, there is a significant inequality in the rates of caesarean section and delivery by skilled health workers favouring the educated. There is also inequality in the provision of antenatal care by skilled provider, although less pronounced (Figure 4)

An increase in the mother's education from each level to the next higher level is associated with an increase in the uptake of interventions. The rate of caesarean section in women with post secondary education is about seven times that of women with no education (35.5% vs. 5%). Furthermore, women with post-secondary education are delivered by skilled providers twice as much as those with no education.

### 3. Place of residence: urban vs. rural

Inequality in the use of interventions is mainly pronounced in antenatal care and delivery by a doctor; antenatal care and delivery in a private facility and delivery by caesarean section. The urban-rural rate ratios for the various maternal services are given in Table 2 below.

**Table 2:** Urban-rural rate-ratios for maternal health interventions

Intervention	Population average rate	Rate-ratio (urban/rural)
Delivery by a skilled provider	81.4	1.3
Antenatal care from a skilled provider	94.6	1.0
Delivery by caesarean section	12.7	3.0
Place of ANC: public facility	90.1	0.9
Place of ANC: private facility	7.9	6.1
ANC provider: doctor	16.1	3.8
ANC provider: nurse/midwife	78.6	0.8
Delivery: doctor	18.6	3.6
Delivery: nurse/midwife	62.8	0.98
Delivery: traditional birth attendant	6.5	0.2
Delivery: relative/other	11.2	0.2
Place of delivery: public	76.3	1.2
Place of delivery: private	4.6	5.9
Place of delivery: Home	18.6	0.2

Note: computed using data from Namibia DHS 2006-07

The rate-ratios that are less than one indicate that those interventions are used more by rural women than the urban ones. For example, a rate-ratio of 0.2 for home delivery indicates that women living in rural areas use home delivery about five times more (i.e. 1/0.2) than those living in urban areas. The same is true for delivery by traditional birth attendants and relatives/others, which are utilized more by women in rural areas. There is no urban-rural differential in antenatal care by skilled providers. However, women in urban areas are delivered by skilled providers 30% more than their rural counterparts.

### 4. Household wealth

One of the limitations of the demographic and health surveys is that they don't collect household income/consumption expenditure data. Therefore, asset indices are computed using the ownership of assets to classify households into wealth quintiles. The wealth quintiles given in the NDHS are used in computing rate-ratios, the concentration index and concentration curve. Although some studies have shown that there is a close relationship between asset indices and consumption expenditure [22], others have discussed some of their limitations and cautioned against making inferences without taking into account the contextual factors [23].

The rate-ratios computed indicate that the uptake of interventions is more among the wealthiest quintile compared to the least wealthy. Table 3 below depicts this information.

**Table 3:** Rate-ratios for maternal health interventions by wealth quintile

Intervention	Population average rate	Rate-ratio (rich/poor)
Delivery by a skilled provider	81.4	1.63
Antenatal care from a skilled provider	94.6	1.06
Delivery by caesarean section	12.7	7.73
Place of ANC: public facility	90.1	0.67
Place of ANC: private facility	7.9	65.2
ANC provider: doctor	16.1	13.4
ANC provider: nurse/midwife	78.6	0.59
Delivery: doctor	18.6	10.6
Delivery: nurse/midwife	62.8	0.86
Delivery: traditional birth attendant	6.5	0.05
Delivery: relative/other	11.2	0.06
Place of delivery: public	76.3	1.3
Place of delivery: private	4.6	213
Place of delivery: Home	18.6	0.06

Note: computed using data from Namibia DHS 2006-07

High levels of socio-economic inequality with pro-rich bias are observed in antenatal care and delivery in private health facilities and antenatal care and delivery by a doctor. The rate-ratios less than one indicate that these interventions are mainly used by the poor. A rate-ratio of 0.06 for home delivery implies that home delivery among the poor is about 16 times that of the economically better-off (i.e. 1/0.06). There is a high level of inequality in delivery at a private health facility. However, it is also important to note that the rich use the public facilities 30% more than the poor for child delivery. This implies that the benefits of subsidized facilities also accrue to the better off.

**Figure 5:**
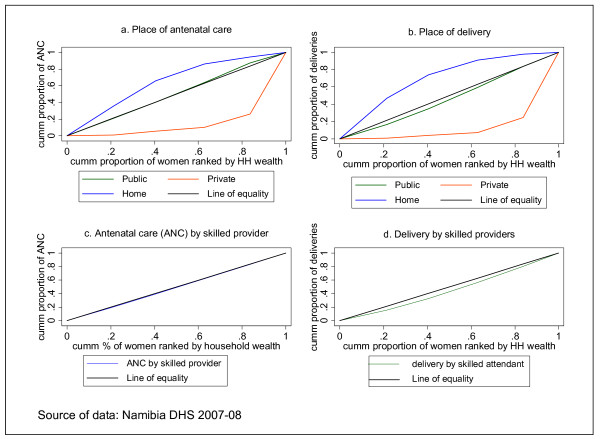
**Concentration curves: various maternal health services**.

Rate-ratios are easy to compute and understand. However, their limitation is that they only take into account the two extreme socio-economic groups - in this case wealth quintiles 1 and 5. This implies that the wealth quintiles in the middle (Quintiles 2, 3 and 4) are disregarded. Hence rate-ratios do not give a composite measure of inequality that takes into consideration all the wealth quintiles. To rectify this flaw of rate-ratios, concentration curves and concentration indices are used. Figure 5 and Table 4 below present the concentration curves and concentration indices for place of antenatal care, place of delivery, antenatal care by skilled provider and delivery by skilled provider.

**Table 4:** Concentration indices for maternal health interventions

Intervention	concentration index	Standard error	95% confidence interval	
			Lower	Upper
Antenatal care by skilled provider	0.0130	0.0045	0.0042	0.0218
delivery by skilled provider	0.0943	0.0248	0.0457	0.1429
Antenatal care: public facility	-0.0541*	0.0397	-0.1319	0.0237
Antenatal care: private facility	0.6435	0.0692	0.5079	0.7791
Antenatal care at home	-0.3409	0.0785	-0.4948	-0.1870
Delivery: public facility	0.0607*	0.0353	-0.0085	0.1299
delivery: private facility	0.6979	0.0903	0.5209	0.8749
Delivery at home	-0.4145	0.1019	-0.6142	-0.2148
Postnatal checkup	0.0835	0.0006	0.0823	0.0847
Delivery by cesarean section	0.3899	0.0662	0.2601	0.5196
Delivery: doctor	0.4326	0.0744	0.2868	0.5784
Delivery: nurse/midwife	-0.0059*	0.0504	-0.1047	0.0929
Delivery: traditional birth attendant	-0.4700	0.1228	-0.7107	-0.2293
Delivery: relative/other	-0.3834	0.0981	-0.5757	-0.1911

* Not significant at p < 0.05

In Figure 5(a), antenatal care in a private facility is seen to be highly inequitable and to the advantage of the rich. The concentration curve is below the line of equality and the furthest compared to the others signifying marked levels of inequity. In contrast the concentration curve for antenatal care at home is above the diagonal implying that home antenatal care is concentrated among the poor. This inequality is significant as corroborated by the concentration index. Antenatal care in public facility does not show any socio-economic gradient, as it almost overlaps with the equality line.

A similar scenario is depicted in Figure 5(b) with respect to delivery in a private facility, i.e. inequity with a pro-rich bias that is statistically significant and home delivery that is significantly more prevalent among the poor. However, delivery in public facilities shows a pro-rich inequality, even though the concentration curve is not far from the line of equality. Hence there is no socio-economic inequality in delivery at public facilities among the wealthy and the poor.

The concentration curve for delivery by skilled health workers has a pro-rich bias; it is more concentrated among the wealthy. The concentration curve for antenatal care by skilled providers seems to overlap with the diagonal. However, the corresponding concentration index indicates that it also has a pro-rich orientation that is statistically significant.

Thus, although the population averages of the indicators, particularly receiving antenatal care from a skilled provider and delivery by skilled health workers are apparently good, the analysis shows that there are serious socio-economic inequalities that are to the advantage of the wealthy.

**Figure 6:**
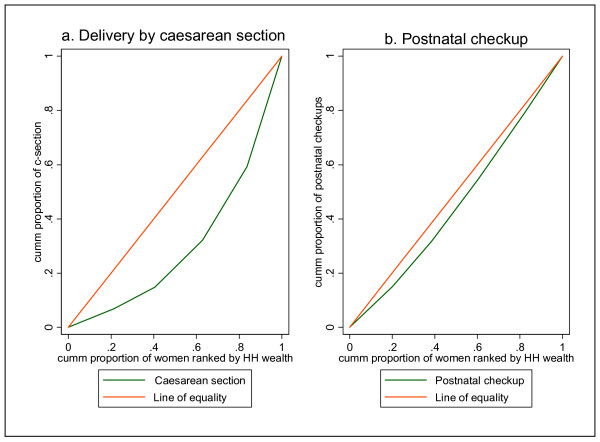
**Concentration curves: delivery by caesarean section and postnatal checkup**.

The situation with caesarean section and postnatal checkup is not different from the above discussed scenarios where there is significantly unequal utilization of the services that favours the wealthy (Figure 6).

Both of the above interventions are highly utilized by the wealthy compared to the poor. It is clearly seen that the concentration index for caesarean section is far below the diagonal line indicating a severe degree of socio-economic inequality in the rate of caesarean section with a bias against the poor. Similarly, postnatal checkup demonstrates the same trend, although the extent of pro-rich inequality is less than that of caesarean section.

## Discussion

This paper has attempted to assess inequities in access to basic maternal health interventions with a view to identifying constraints that may impede progress towards the MDG 5 target of reducing maternal mortality. The findings demonstrate that in the absence of targeted interventions, the achievement of MDG 5 target will be difficult.

The findings indicate significant spatial inequalities in the utilization of basic maternal health interventions. This is in line with previous studies [2,5]. Use of skilled providers for child delivery services is much below the national average in three regions: Kavango, Kunene and Ohangwena. The rate in other four regions is also less than the national average of 81.4%, although it is better than the three regions mentioned above. Thus, it is evident that resource allocation decisions based on the national average figures may not lead to appropriate targeting of the scarce maternal health resources and that the regions who need it more may not receive what is due to them to achieve the target of reducing maternal mortality.

The inverse relationship between delivery by skilled attendants and maternal mortality has been well-established [24]. Thus, to reduce the increasing levels of maternal mortality ratio and expedite progress towards the MDG 5 target, it is necessary to focus all efforts and interventions towards those regions that have worse-off indicators in terms of access to maternal health interventions.

The regions that have lower coverage of the basic maternal interventions are also the ones that have low human development index. Hence, to improve maternal health and promote equity, it is necessary to launch a multi-sectoral action that also addresses the social determinants of health such as poverty and levels of education in line with the recommendations of the Commission on Social Determinants of Health.

The rate of caesarean section in the region with the highest rate (Khomas) is 13 times more than that of Caprivi with the lowest rate. Three regions - Caprivi, Kunene and Ohangwena - have rates that are less than 5%. In contrast three regions - Erongo, Khomas and Omaheke - have caesarean section rates well above 15%. This indicates that there are regions of under-coverage as well as region where there is over-provision of caesarean section. Although there is a debate, a population-based rate of 5-15% has been considered as the acceptable level for cesarean section to ensure the best outcomes for mothers and children [25]. The proportion of deliveries by cesarean sections in a geographical area is a measure of access to and use of obstetric emergency care for averting maternal and neonatal deaths. Therefore, it is evident that there is limited access to comprehensive emergency obstetric care in a third of the regions mentioned that requires urgent action.

Education-related inequalities in the rate of cesarean section and delivery by skilled providers are more pronounced that those of antenatal care by skilled providers. There is an excess utilization of cesarean section among women with post-secondary education. This is more than twice the threshold for the acceptable limit of cesarean section. Almost all women with post-secondary education are delivered by skilled providers compared to about half of those with no education. Women who are primary school complete use skilled providers for delivery 60% more than those with no education. Women's education influences health outcomes through a variety of channels including health-seeking behaviors and earning opportunities. In Namibia, income poverty has a very wide variability by educational groupings. The 2003/2004 household income and expenditure survey indicates that while the incidence of poverty among those without formal education is 41.4%, it is only 0.5% among those with tertiary education [13]. These may influence the demand for maternal health services. As stated above, it is therefore necessary to improve mother's level of education and its correlates in order to bridge the inequity and improve uptake of essential maternal health interventions.

A significant rural-urban inequality in the use of interventions is also observed. Delivery by cesarean section, antenatal care in a private facility, antenatal care by a doctor, delivery by a doctor, delivery in a private facility and delivery by a skilled attendant demonstrate inequalities in favour of the rich. Use of private facilities and doctors for antenatal care and delivery and cesarean section is lowest in rural areas. In contrast, rural women use more of delivery by traditional birth attendant, delivery by a relative/other, delivery at home. However, antenatal care by a skilled provider does not show any significant inequalities between urban and rural areas, which may indicate that although mothers access health facilities for ANC, only a proportion of these offer delivery services. Women in urban areas also make more use of delivery services in public facilities compared to those in rural areas. This may perhaps be linked to the levels of poverty and educational status, which also manifest rural-urban disparity.

The concentration curves and indices indicate significant pro-wealthy inequalities in antenatal care and delivery by skilled providers; delivery in public and private health care facilities; delivery by cesarean section; and post-natal checkup. In contrast concentration curves and indices for antenatal care and delivery at home have a pro-poor bias.

Addressing the poor-rich inequalities in delivery care by skilled attendants is essential for achieving the MDGs for maternal health [3]. However, the low rate of delivery by skilled personnel in some of the regions, among the less educated, rural areas and the less wealthy is of concern, as the groups where most of the improvements in maternal health are expected have a limited access. This will lead to a slow progress in halting the current trend of increasing maternal mortality ratio and achievement of the MDG 5 targets.

The case of cesarean section is also another area of concern, as conditions that require comprehensive emergency obstetric care are major causes of maternal mortality. Under-provision among the poor, rural areas, less educated and regions with low human development index and over-provision among the wealthiest, more educated, urban areas and regions with high human development signal the need for re-allocation and targeting of the available resources in order to make a significant contribution to the reduction of maternal mortality in line with the MDG 5 target.

The inequities observed in this study may be explained by demand and supply side factors [3,23]. For example mother's education may affect health-seeking behaviour and together with household wealth may also constrain the demand for services. The NDHS 2006-07 indicates that almost all women pay for delivery mainly in cash and to a lesser extent in kind. About 86% of women who had live births in the five years preceding the survey paid in cash [8]. For the majority (85%), the payment was less than 50 Namibian dollars (about US$ 7 at the then prevailing exchange rate). Adding to this the indirect costs that the women and those accompanying them are likely to incur (e.g. transport cost), payment for deliveries could be a barrier to use of delivery services by trained providers for those poorest segments of the population. It is therefore worthwhile to revisit the policy of charging women for delivery services and possibly make blanket exemptions in those regions where the poverty levels/HDI are the lowest in order to increase uptake of interventions caused by demand side factors. It should also be noted that the revenue generated from these payments for delivery is a very negligible fraction of the total government expenditure on health [14] and that it doesn't play a significant role in terms of revenue generation.

The increasing trend in the maternal mortality ratio may also be related to the supply of services. In 2005/06, only 11.8% of health facilities provided comprehensive emergency obstetric care, which in addition to those services under basic emergency obstetric care, includes blood transfusion and the provision of caesarean section. Furthermore, no health centres provided basic emergency obstetric care [26].

With per capita expenditure on health of US$ 276 in 2006, the country is in a better off position than most countries in the African region, where the average per capita expenditure on health in 2006 was US$ 58 [14]. Therefore it is necessary to address possible allocative inefficiency, where resources may be allocated in purchasing the non-optimal mix of inputs and/or producing the non-optimal mix of outputs. The non-provision of basic emergency obstetric care at the health centre level may, among other things, imply the presence of regulatory frameworks that do not allow junior level health workers to provide the basic services (signal functions). To halt the upward trend in maternal mortality ratio, bottlenecks related to policy and regulatory frameworks have to be addressed and issues of possible task-shifting be explored.

Inequality in the distribution of health care inputs is also one of the important factors that may contribute to the limited access to essential maternal health services, consequently leading to increase in the maternal mortality ratio. The Namibia health and social services system review indicated a health worker density of 3 per 1,000 population [26], which is well above the estimated minimum level of health workforce density of 2.5 per 1000 population required to achieve 80% coverage of immunization and delivery by skilled attendants [27]. However, a breakdown of this national figure indicates that while the health workforce density in the private sector was 8 per 1000 (high density), in the public sector it was only 2 (low density), which is below the threshold stated above.

Explaining inequities to maternal health interventions in terms of demand and supply fits well with the three delays model [28]. This model proposes three barriers to accessing maternal health services: (i) delay in decision to seek care; (ii) delay in getting to the facility; and (iii) delay in getting the appropriate care once at the facility. The first two delays are demand-side barriers, which may be affected by mother's education, household wealth and community-level factors such as the levels of poverty, which also have a bearing on the intra-household resource allocation and inequities. It can be discerned that the causes of inequities in the utilization of maternal health interventions are those that may explain the delays model. However, this study recommends that the causes of the inequities be identified using a decomposable concentration index [20] in order to target resources at the root causes of the inequities.

In the presence of inequities, achievement of the MDG and other national and international targets becomes elusive. The segment of society that has more need is left out, thus impeding progress towards the cherished goals. Some of the access gradients observed, including educational status and geographical location, lie outside the health sector. Hence, addressing inequities in access to maternal health services should not only be seen as a health systems issue. The social determinants of health have to be tackled through multi-sectoral approaches in line with the principles of Primary Health Care [29] and the recommendations of the Commission on Social Determinants of Health [18]. The above-mentioned factors that are outside the health sector can be tackled through action points within the three overarching recommendations of the Commission on Social Determinants of Health [18]. To this end, it is necessary to assess the contribution of each of the above determinants to the overall inequality in access to the various maternal health interventions.

## Competing interests

The authors declare that they have no competing interests.

## Authors' contributions

EZ designed the study, performed the analysis and drafted the report; PT, OW, JK, CM and TM participated in the write-up and revision of the manuscript. All authors read and approved the final manuscript.
